# Spectroscopic Detection of Cyano-Cyclopentadiene Ions as Dissociation Products upon Ionization of Aniline

**DOI:** 10.1021/acs.jpca.2c01429

**Published:** 2022-05-05

**Authors:** Daniël B. Rap, Tom J. H. H. van Boxtel, Britta Redlich, Sandra Brünken

**Affiliations:** Radboud University, Institute for Molecules and Materials, FELIX Laboratory, Toernooiveld 7, 6525 ED Nijmegen, The Netherlands

## Abstract

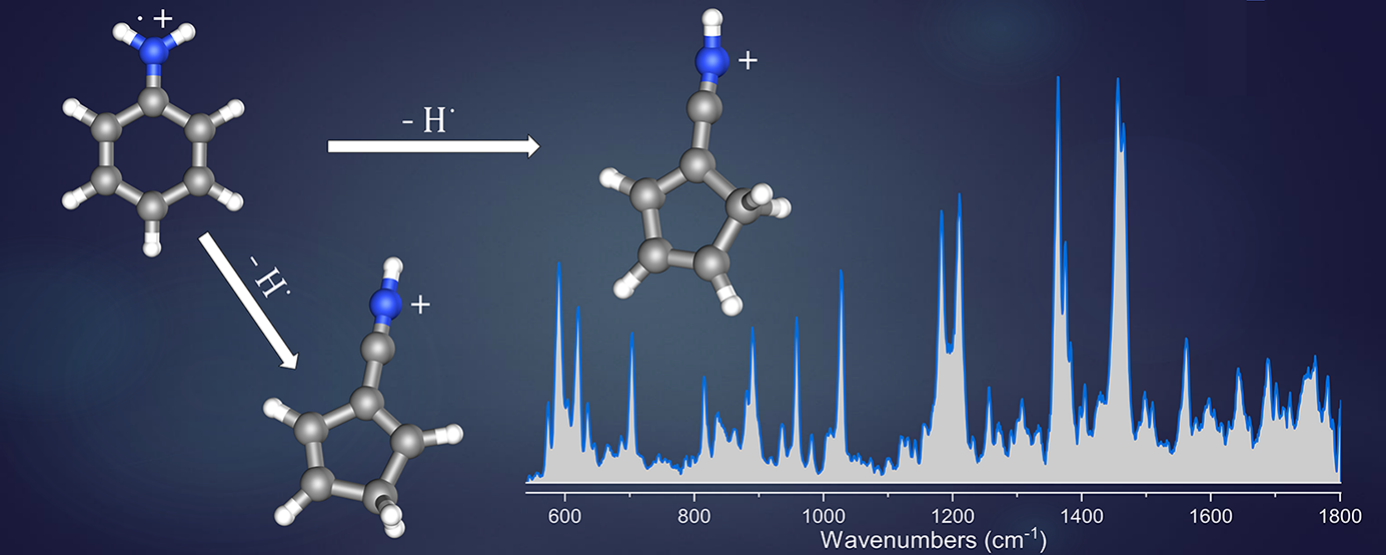

The H-loss products (C_6_H_6_N^+^) from the dissociative ionization of aniline (C_6_H_7_N) have been studied by infrared predissociation spectroscopy in a cryogenic ion trap instrument at the free electron laser for infrared experiments (FELIX) laboratory. Broadband and narrow line width vibrational spectra in the spectral fingerprint region of 550–1800 cm^–1^ have been recorded. The comparison to calculated spectra of the potential isomeric structures of the fragment ions reveals that the dominant fragments are five-membered cyano-cyclopentadiene ions. Computed C_6_H_7_N^•+^ potential energy surfaces suggest that the dissociation path leading to H loss starts with an isomerization process, following a similar trajectory as the one leading to HNC loss. The possible presence of cyano-cyclopentadiene ions and related five-membered ring species in Titan’s atmosphere and the interstellar medium are discussed.

## Introduction

More than 260 molecular species have been observed in different astronomical environments, thereby contributing to the long-standing astronomical questions of the existence and possible formation pathways of complex (organic) molecules in space.^1^ Recently, multiple molecules were detected with radio-astronomical observations that can be associated with the carriers of the aromatic infrared bands (AIBs) and diffuse interstellar bands (DIBs), which are spectral fingerprints observed in the infrared and visible region that point toward the presence of large organic molecules such as polycyclic aromatic hydrocarbons (PAHs)^2^ and fullerenes.^3,4^ Among these molecules are aromatic and cyclic species such as benzonitrile,^5^ cyanonaphthalenes,^6^ indene,^7,8^ cyclopentadiene,^7^ and cyano-cyclopentadienes (cyano-CPDs).^9,10^ Complex organic molecules have also been detected in the atmospheres of Jupiter, Saturn,^11^ and Titan^12,13^ and on other bodies such as meteorites^14^ and comets^15^ within our own Solar System, illustrating that large organic molecules are omnipresent in the universe. An extensive organic chemistry has been observed in the atmosphere of Titan, Saturn’s largest moon, by the Cassini spacecraft where signatures of important chemical building blocks such as protonated pyridine (C_5_H_5_NH^+^) and protonated aniline (C_6_H_7_NH^+^) and their closed shell cations, C_5_H_4_N^+^ and C_6_H_6_N^+^, respectively, have been detected by mass spectrometry.^12,16,17^ Solar radiation can initiate the chemistry by photoionization and photodissociation of N_2_ and CH_4_, which ultimately lead to the formation of a thick organic haze in the atmosphere of Titan. Within these chemical networks, multiple neutral–neutral and ion–neutral molecular reactions and fragmentation pathways of organic molecules are involved and coexist.^17,18^ To disentangle the chemistry, experimental studies on the exact fragmentation and reaction pathways are important for chemical network models and to aid the discovery of new molecules in the different physical and chemical regions of space such as the interstellar medium (ISM) and planetary atmospheres.

The fragmentation pathways of even relatively small and simple molecules can already be challenging to investigate using both quantum chemistry and laboratory experiments. Many different pathways can coexist and be accessible at a given internal energy. Mass spectrometry can provide the fragmentation pattern, i.e., the relative abundance of different fragment masses from which one can elucidate the parent structure. However, precise structural information cannot be gained using mass spectrometry only. When spectroscopic methods are added, isomeric structures along the fragmentation trajectory and of the end products can be structurally distinguished. For example, the loss of acetylene and hydrogen by the PAH naphthalene has been studied using a combination of techniques, including imaging Photoelectron Photoion Coincidence (iPEPICO) spectroscopy and tandem mass spectrometry^19^ and two-photon dissociative ionization with subsequent infrared multiphoton dissociation (IRMPD) spectroscopy.^20^ In the latter study, the structure of the acetylene-loss fragment was unambiguously determined by infrared spectroscopy to be the five-membered bicyclic pentalene^•+^ cation. Similarly, dissociative ionization by electron impact and subsequent infrared predissociation (IRPD) spectroscopy has revealed the structure of the H_2_-loss fragment of pyrene.^21^ Other techniques, mainly targeting neutral molecules, involve electrical discharges to induce fragmentation and IR-UV ion-dip spectroscopy^22^ or broadband cavity enhanced Fourier-transform (FT) microwave spectroscopy^23,24^ for structure elucidation. These combined approaches, together with quantum chemical calculations of the potential energy surface (PES), can give a clear understanding of the fragmentation pathways of diverse molecular systems.

Photodissociation studies on the H loss of neutral aniline has shown cleavage of the N–H bond, forming the anilino radical as the dominant product.^25,26^ Further decomposition of this neutral anilino radical, also produced in the ozonation of aniline, has been studied using computational methods.^27^ The structures of the fragment ions from dissociative ionization of aniline are less clear. Earlier studies using multiphoton mass spectrometry have identified multiple metastable ion decay channels of the aniline radical cation, including H and HNC loss.^28^ More recent ionization and dissociation studies using vacuum ultraviolet (VUV) radiation have shown that hydrogen atom transfer plays an important role in the cleavage of bonds in aniline ions,^29^ highlighting the importance to consider isomerization prior to dissociation. More details on the potential energy surface for the dissociation of the aniline radical cation have been provided by Choe et al.,^30^ who performed density functional theory and Rice–Ramsperger–Kassel–Marcus (RRKM) calculations. The HNC fragmentation pathway has been calculated to occur via ring opening and reclosure steps involving five-membered ring intermediate structures. Additionally, at high internal energies, competing pathways for H loss were identified via direct N–H bond cleavage or the formation of a seven-membered ring.

Here, we study the dissociative ionization pathways of the nitrogen containing aromatic molecule aniline (aminobenzene), the simplest aromatic amine, upon hydrogen atom (H) loss and provide a spectroscopic identification of the ionic H-loss fragment (C_6_H_6_N^+^). We apply sensitive infrared predissociation (IRPD) action spectroscopy in a cryogenic ion trap instrument^31^ coupled to the free electron laser for infrared experiments (FELIX)^32^ to investigate the mass-selected ionic fragments formed upon dissociative ionization of neutral aniline, a method that we have previously applied to identify the HNC- and CO-loss fragment ion of aniline and phenol,^33^ respectively, as well as the H-loss fragment ions of larger aromatic species.^21,34^ The observed structures provide additional information on the isomerization and fragmentation pathways of aniline compared to previous theoretical and experimental studies. The spectroscopic identification of the different fragmentation pathways of aniline can point toward new astronomically relevant molecules that are likely to exist in both Titan’s atmosphere and cold molecular clouds.

## Experimental and Computational Methods

### FELion Cryogenic 22-Pole Ion Trap Instrument at the FELIX Laboratory

The experiments were performed using the cryogenic ion trap beam station at the FELIX laboratory, which has been described in more detail elsewhere.^31^ In general, a liquid aniline sample (99.5%, Sigma-Aldrich) was evaporated and ionized by electron impact (EI) with 15–50 eV electrons and subsequently fragmented. Two different ion sources, a Gerlich-type storage ion source^35^ and a direct EI source, were used. The experimental infrared spectrum of the fragment ions was obtained using infrared predissociation (IRPD) spectroscopy. The ionic H-loss fragment with *m*/*z* 92 (C_6_H_6_N^+^) was mass-selected by a quadrupole mass selector and stored in the 22-pole ion trap, which was kept at a temperature of 6 K. The ions were kinetically cooled close to this temperature by a high-density pulse of a 2:1 He/Ne mixture. Complexes of neon and the ion of interest were formed and stored in the cryogenically cooled 22-pole ion trap prior to the spectroscopic investigations. The infrared fingerprint of the C_6_H_6_N^+^ fragment ion was obtained by dissociation of the weak ion-rare gas bond of C_6_H_6_N^+^–Ne upon resonant single photon excitation using the intense and tunable free-electron laser FELIX.^32^ By detecting the number of C_6_H_6_N^+^–Ne complexes as a function of the wavelength, the infrared fingerprint region between 550 and 1800 cm^–1^ was measured, and the comparison with characteristic vibrational modes obtained from quantum chemical calculations was enabled. The influence of the rare gas tag on the positions of the vibrational bands has been investigated in multiple other studies and is shown to introduce only small shifts on the order of several cm^–1^.^36−39^ The IRPD spectrum of the complex can thus be regarded as a good proxy for that of the bare cation that will not influence the assignment of the spectrum to a specific isomeric structure. The wavelength was calibrated with an infrared spectrum analyzer, and the intensity (*I*) was normalized for the laser pulse power (*E*), number of laser pulses (*N*) and the photon energy (*h*ν) using the following equation:
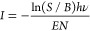
1with *S* being the number of C_6_H_6_N^+^–Ne complexes as a function of the wavelength and *B* being the number of baseline C_6_H_6_N^+^–Ne complexes. Multiple scans have been performed in the 550–1800 cm^–1^ range and were subsequently averaged with a typical bandwidth of 0.5% fwhm and 10 Hz macropulse power up to 30 mJ. The IRPD method allowed one to perform so-called saturation depletion measurements where specific vibrational bands of one isomeric species were fully depleted until saturation to estimate the abundance of this isomer.^37,39,40^

### Quantum Chemical Calculations

To interpret the experimental infrared spectrum of the H-loss fragment, potential structures suggested in the literature were considered and treated by density functional theory (DFT) using Gaussian 16.^41^ The B3LYP functional including dispersion correction GD3 in combination with the N07D basis set was used to optimize the molecular structures, to find their energetic minima, and to perform calculations of the harmonic and fundamental vibrational frequencies.^42−44^ This functional/basis set combination has been shown to accurately predict vibrational mode frequencies of (polycyclic) aromatic molecules.^43,45,46^ The anharmonic fundamental frequencies, overtone, and combination bands were calculated using the VPT2 functionality of Gaussian 16. To investigate the potential energy surface of the aniline fragmentation, transition states discussed in the literature and additional transition states between the energetic minima were first calculated and verified using intrinsic reaction coordinate (IRC) calculations at the B3LYP-GD3/N07D level of theory. To obtain more accurate electronic energies, the minima and transitions states were further optimized using the CBS-QB3 method.^47,48^ The energies of the structures were corrected for the zero-point vibrational energy before comparison.

## Results and Discussion

### Fragmentation Mass Spectrometry

**Figure 1:**
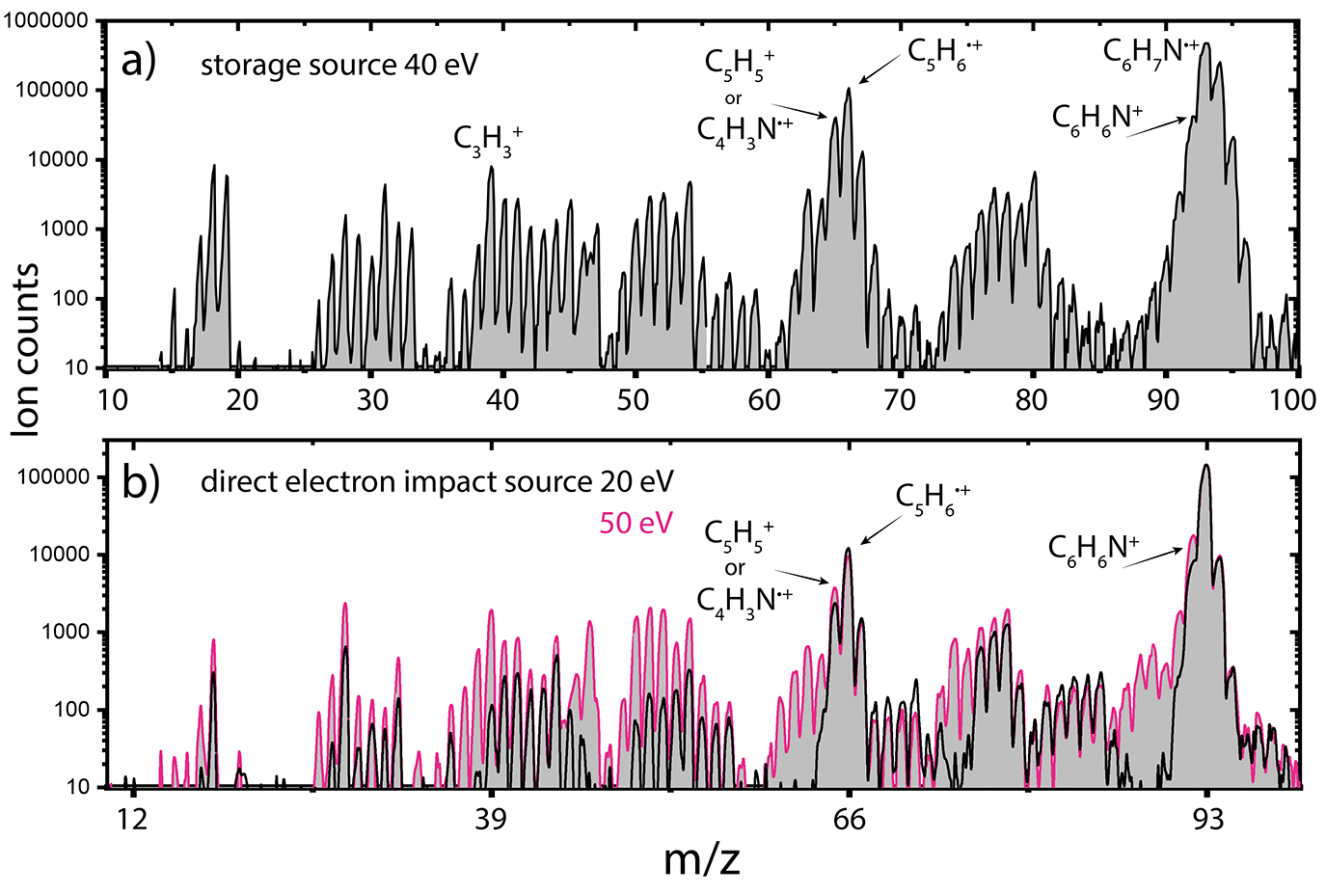
Experimental mass spectra showing the ionization and fragmentation of aniline in (a) the storage ion source at 40 eV and (b) the direct electron impact source at electron energies of 20 eV^33^ (black) and 50 eV (pink). The most important fragmentation channels C_6_H_6_N^+^ (*m*/*z* 92, H loss), C_5_H_6_^•+^ (*m*/*z* 66, HNC loss), and C_5_H_5_^+^ or C_4_H_3_N^•+^ (*m*/*z* 65, HNCH or C_2_H_4_ loss) are labeled.

To determine the structure of the H-loss fragment, we used a combination of dissociative ionization by electron impact, mass spectrometry, and infrared action spectroscopy to measure the experimental infrared spectrum of the C_6_H_6_N^+^ ion. The fragment ions were formed upon electron impact on aniline vapor, leading to the ionization of aniline (ionization energy: 7.72 eV^49^), forming the radical cation C_6_H_7_N^•+^ at *m*/*z* 93, and multiple fragmentation products including *m*/*z* 92 (H loss), *m*/*z* 66 (HNC loss), and *m*/*z* 65 (HNCH or C_2_H_4_ loss) as shown for a high electron energy fragmentation spectrum at 40 eV in Figure 1a.

The appearance energy of *m*/*z* 66 (11–13 eV^50,51^) is well below the energy provided by the electrons making this fragmentation channel accessible. Theoretical calculations on the branching ratio of the HNC and H-loss pathways show that the loss of HNC is dominant over H loss but that the ratio decreases upon increasing internal energy of the ion.^30^ A comparison between two different electron energies used in the direct ion source supports this and shows an increasing dominance of the H-loss fragment upon high electron energies of 50 eV when compared to the 20 eV fragmentation mass spectrum (Figure 1b). Additionally, an increase in the fragmentation channel at *m*/*z* 65 is seen, hinting at subsequent H loss from the *m*/*z* 66 fragment structure. Also, many small fragments are present in the 50 eV fragmentation spectrum similar to what is observed in the storage source spectrum at 40 eV. We can regard the fragmentation patterns obtained in the two different ion sources at these higher energies as similar, though we generally observe a higher ion yield and a more efficient formation of protonated species using the storage source due to secondary reactions.

The fragmentation pathway toward *m*/*z* 66 has been studied extensively using theoretical methods^30^ and experimental methods.^28,29,52,53^ Lifshitz et al.^52^ established that the fragmentation occurred to the cyclopentadiene radical cation with the ejection of neutral hydrogen isocyanide (HNC). Using cryogenic infrared messenger spectroscopy, the structures of the aniline parent ion (*m*/*z* 93) and the HNC fragment (*m*/*z* 66), formed at an electron energy of 20 eV, have been investigated and structurally assigned to the canonical aniline^•+^ radical cation and the cyclopentadiene^•+^ radical cation structure, respectively.^33^ The same experimental approach is used here to elucidate the experimental structure of the H-loss product. The spectroscopic measurements were performed with the storage ion source using an electron impact ionization with 15 eV electrons. After ionization of aniline, ∼7 eV (675 kJ/mol) of additional energy can be available as internal energy in the molecule to induce the isomerization and fragmentation processes. This was sufficient to observe the H-loss fragment and corresponds to similar fragmentation conditions as those for the earlier HNC-loss fragment study.

### Infrared Fingerprinting

To obtain the infrared fingerprint spectrum, complexes of C_6_H_6_N^+^ and neon were formed in the trap and irradiated with the tunable infrared laser light of FELIX in the molecular fingerprint region. The C_6_H_6_N^+^ molecules showed a significant binding affinity for neon atoms resulting in the formation of C_6_H_6_N^+^–Ne_*n*_ complexes with *n* up to or more than six (Figure S1). Note that *m*/*z* 93 is not completely filtered out before trapping, but this does not influence the spectroscopic measurements as we have only monitored the depletion of the C_6_H_6_N^+^–Ne (*m*/*z* 112) channel. Likewise, no effects of higher Ne_*n*_ complexes on the C_6_H_6_N^+^–Ne channel were observed.

**Figure 2:**
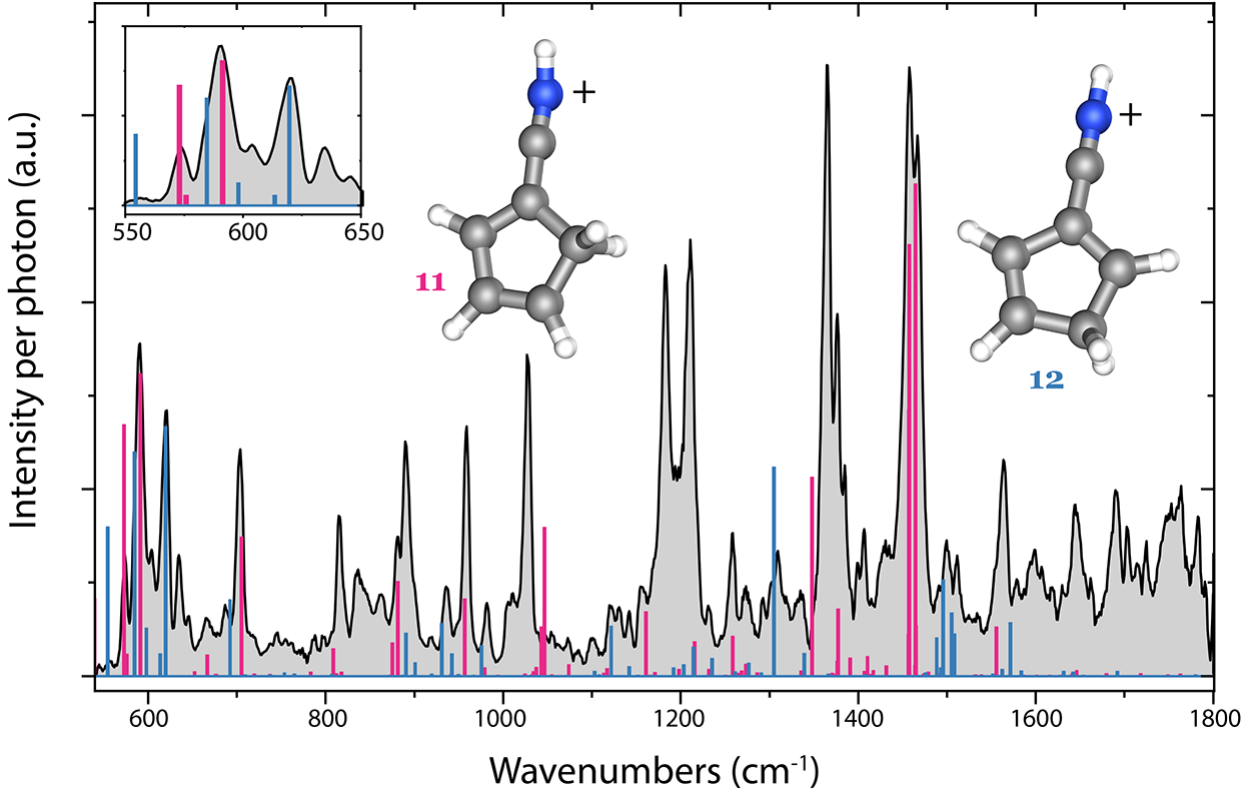
Experimental infrared predissociation spectrum of C_6_H_6_N^+^–Ne (gray) and calculated anharmonic infrared spectra of H-1-cyano-CPD^+^ (pink) and H-2-cyano-CPD^+^ (blue) plotted with sticks. The inset shows a close-up of the lower wavenumber region. The B3LYP-GD3/N07D level of theory was used to optimize the geometries of the two isomers and calculate the vibrational frequencies.

**Figure 3:**
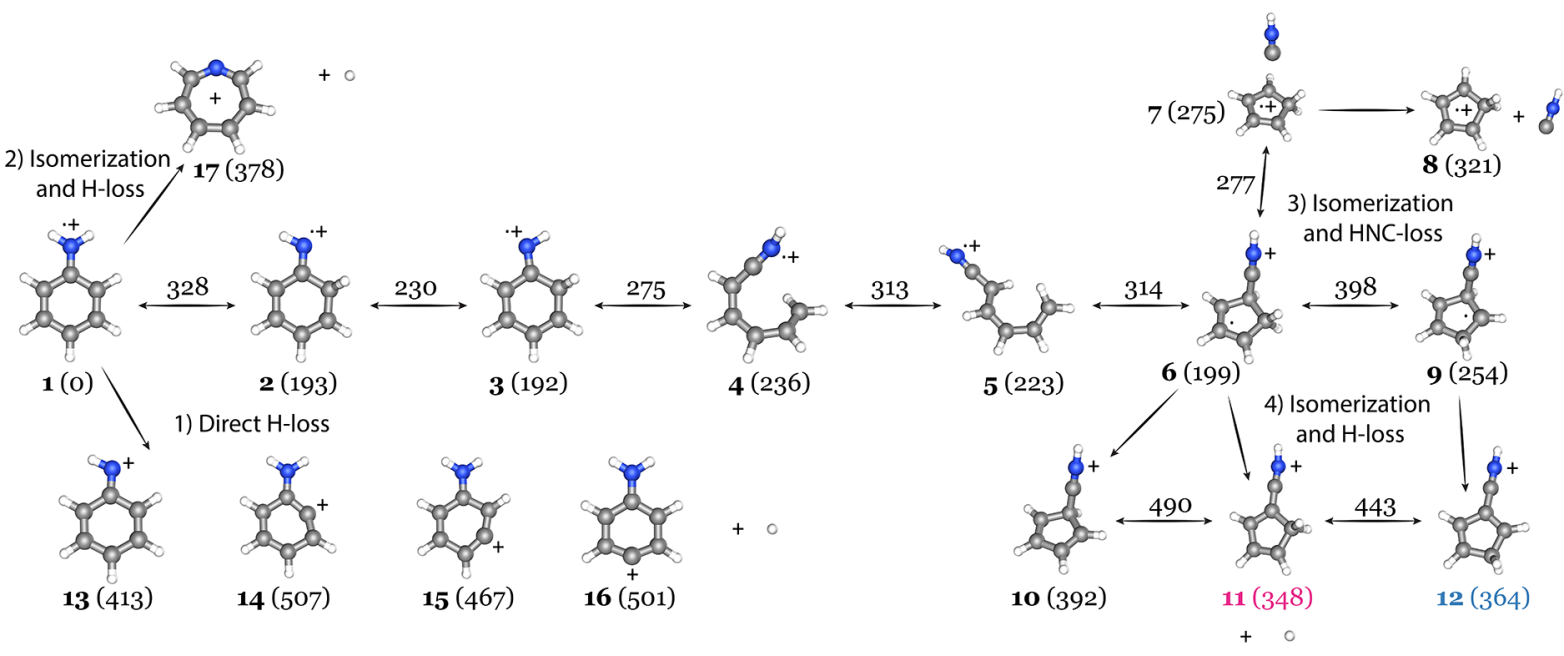
Overview of the different fragmentation pathways of aniline^•+^ via (pathway 1) direct H loss, (pathway 3) isomerization and HNC loss, and (pathways 2 and 4) isomerization and H loss. The potential energy surface was evaluated using the CBS-QB3 method. The zero-point corrected electronic energies are given relative to aniline^•+^ (**1**) in kJ/mol between parentheses for minima and above the arrows for transitions states, respectively. The structures observed here are colored in pink and blue according to the structures **11** and **12** from Figure 2, respectively.

**Figure 4:**
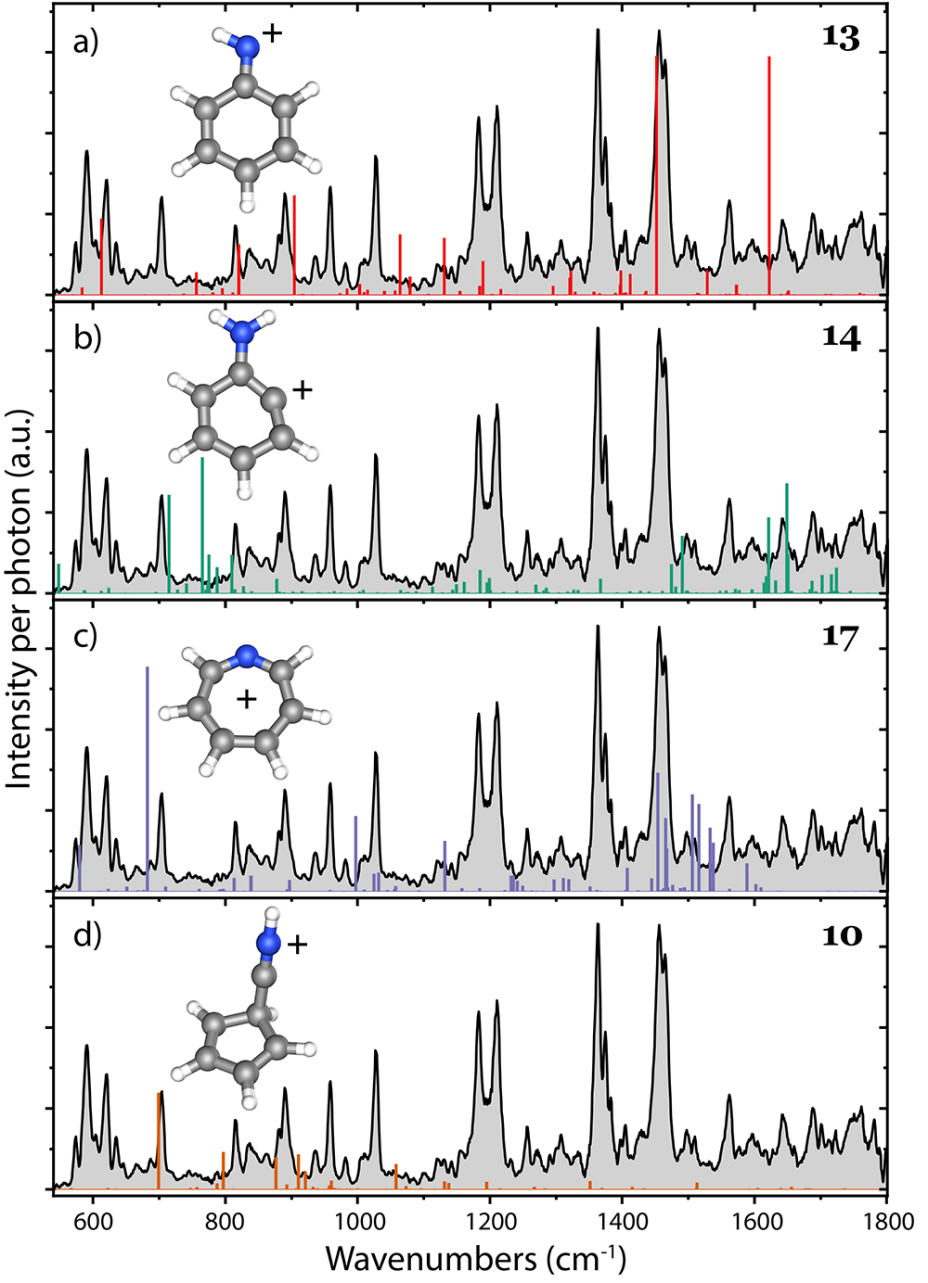
Experimental infrared spectrum of *m*/*z* 92 (gray) and comparison with the calculated anharmonic frequencies of (a) anilino^+^ (red), (b) 1-dehydro-aniline^+^ (green), (c) azatropylium^+^ (blue), and (d) H-5-cyano-CPD^+^ (brown).

The experimental infrared fingerprint of C_6_H_6_N^+^ is shown in Figure 2. A comparison is made with two calculated anharmonic infrared spectra of protonated 1-cyano-1,3-cyclopentadiene (H-1-cyano-CPD^+^, C1) (**11**) and protonated 2-cyano-1,3-cyclopentadiene (H-2-cyano-CPD^+^, Cs) (**12**). The numbers in the figure and throughout the text correspond to the structures on the PES as shown in Figure 3. Characteristic features of both species can be found in the experimental spectrum, whereas other possible and energetically low-lying structures depicted in Figure 4 show no clear match, as will be discussed in more detail later. A strong doublet around 1460 cm^–1^ can only be attributed to H-1-cyano-CPD^+^. This vibration is characterized by the C=C stretches of the five-membered ring. Saturation depletion scans performed on the 703 and 816 cm^–1^ bands, belonging to H-1-cyano-CPD^+^, give an estimate of 66–78% abundance of this isomer (Figures S2 and S3). A strong feature at 620 cm^–1^ can be assigned to the other isomer, H-2-cyano-CPD^+^, presenting the overtone of the N–H out-of-plane bending mode. Individual scans indicate a lower limit of ∼15–25% on the abundance for the minor isomer based on the features at around 1300 and 1500 cm^–1^ that are exclusively present due to the H-2-cyano-CPD^+^ isomer. Both isomeric species contribute to the band observed at around 590 cm^–1^, which is highlighted using the inset in Figure 2. Two strong features at 1182 and 1210 cm^–1^ are not explicitly explained by the anharmonic calculations of either isomer but can be ascribed to the overtone of the 590 cm^–1^ feature calculated for the H-1-cyano-CPD^+^ isomer and the combination band of the 590 and 620 cm^–1^ features calculated for the H-2-cyano-CPD^+^ isomer. Both features show similar vibrational modes: 590 cm^–1^ is the in-plane N–H wagging (slightly offset from the plane), and the band at 620 cm^–1^ depicts the N–H out-of-plane (OOP) bending mode. Anharmonic treatment using the VPT2 method using Gaussian generally fails to predict these X-H OOP bending modes and their combination bands and overtones correctly. Other experiments have shown a similar effect for ethynyl group C–H OOP bending modes, where intense overtones of these vibrations around 1200 cm^–1^ are observed in the experimental spectrum but not predicted by theory.^22^

### Potential Energy Surface of Fragmentation

The observed structures are clearly different from those previously proposed. Choe et al.^30^ suggested the formation of the anilino^+^ cation (**13**) where the loss of H occurred from the NH_2_ group and another pathway that involves the isomerization toward the seven-membered ring azatropylium^+^ (**17**) on the basis of quantum chemical and RRKM calculations. To explain the observed structures for the H-loss product of aniline and the rejection of other plausible structures, the two assigned H-cyano-CPD^+^ structures and their relative energies have been computed here and added to the potential energy surface of C_6_H_7_N^•+^ as shown in Figure 3 (extension to Choe et al.^30^).

The direct H-loss products from pathway 1 leading to the anilino^+^ (**13**) and 1-, 2-, and 3-dehydroaniline^+^ (**14**–**16**) are relatively high in energy compared to the pathways 2–4 that involve isomerization prior to the loss of a hydrogen atom. Pathway 2 is described in more detail by Choe et al.^30,54^ and yields the azatropylium^+^ (**17**) with a lower relative energy of 378 kJ/mol compared to the anilino^+^ species that exists 413 kJ/mol above the entrance energy. Earlier, Rinehart et al.^53^ proposed that the H-loss fragment is likely rearranged and would correspond to the azatropylium^+^ ion. It was also mentioned that the parent ion (*m*/*z* 93) was almost entirely unrearranged, as recently confirmed by infrared action spectroscopy on the ion,^33^ and furthermore that isomerization of the aniline^•+^ always leads to dissociation if sufficient energy is available for the fragmentation channel. Pathway 3 includes the isomerization and fragmentation to C_5_H_6_^•+^ upon the loss of HNC, where the product ion has been experimentally observed under similar ionization conditions as in this study and assigned to the cyclopentadiene^•+^ radical cation (**8**).^33^ On this isomerization pathway, the H-loss branch has been calculated from the intermediate structure (**6**) resulting in (**10**). Structure **10** is calculated to be 392 kJ/mol higher with respect to the aniline^•+^, thereby higher than azatropylium^+^ but slightly lower than the anilino^+^ ion.

By calculating the infrared fingerprint spectra of the other H-loss products, we can compare those to the experimental spectrum and evaluate if these isomers are present in small abundance in this experiment. The comparison with the most relevant H-loss structures is shown in Figure 4.

The anilino^+^ isomer (**13**) can be rejected on the basis of the strong band predicted at around ∼1620 cm^–1^ (C=C ring stretching vibrations), which is clearly missing in the experimental spectrum (Figure 4a). The same can be concluded for 1-dehydroaniline^+^ (**14**) and azatropylium^+^ (**17**) on the basis of the strong vibrational modes predicted at around ∼760 cm^–1^ (overtone, NH_2_ OOP wagging mode) and 680 cm^–1^ (seven-membered ring C–H OOP bending mode), which are not present in the experimental spectrum (Figure 4b,c). We cannot exclude a small contribution of the protonated 5-cyano-1,3-cyclopentadiene^+^ (H-5-cyano-CPD^+^) (**10**), as many vibrational modes coincide with experimental features. The rejection of these other isomers confirms/strengthens the assignment of the two protonated 1- and 2-cyano-CPD^+^ isomers.

To understand the formation of these species, additional H-loss pathways need to be included in the PES. A fragmentation pathway from the intermediate (**6**), where the hydrogen atom is lost from the cyano-substituted carbon atom, has been found leading to **11**, which has the lowest energy (348 kJ/mol) among the H-loss products. On the basis of the spectroscopic characterization in the previous section, **11** is the dominant isomer observed for C_6_H_6_N^+^. Also, upon hydrogen migration, structure **6** can interconvert to **9**, and subsequent H-loss yields the product structure **12**, also with a relatively low energy of 364 kJ/mol, which has been assigned to be the other isomer for C_6_H_6_N^+^. However, the barrier for this hydrogen migration is quite significant with 398 kJ/mol and might explain why the second isomer exists in lower abundance. The H-5-cyano-CPD^+^ structure (**10**) is slightly higher in energy (392 kJ/mol) but cannot be completely ruled out on the basis of the spectroscopic similarities (Figure 4).

The loss of a hydrogen atom via a direct bond breaking mechanism (pathway 1) is calculated to be higher in energy compared to the isomerization and subsequent fragmentation pathways 2–4. On the basis of the spectroscopic measurements, no evidence of fragment structures from pathway 1 has been found. However, also no signatures of azatropylium^+^ (**17**), one of the lower energy species, have been detected, thereby lacking evidence for pathway 2. On the basis of the significant abundance of the *m*/*z* 66 fragment (HNC loss), also at low electron energies of 20 eV (Figure 1b), we can argue that the isomerization and fragmentation via pathway 3 proceeds efficiently. Moreover, the final HNC-loss fragment CPD^•+^ (**8**) at 321 kJ/mol is lower in energy than the different H-loss fragments. At higher electron energies, the branching ratio between HNC and H loss changes and more of *m*/*z* 92 is formed (Figure 1b). On the basis of the experimentally determined structures, we suggest a branched fragmentation pathway starting within the isomerization pathway 3 that leads to *m*/*z* 66, where a H-loss fragment channel (pathway 4) opens to form the H-cyano-CPD^+^ isomers (**11**, **12**) from the five-membered ring intermediate structure (**6**). The first step of the isomerization process, the ring contraction, has been previously observed for the thermal processing of the structural analog molecule phenylnitrene (C_6_H_5_N) where a similar rearrangement to neutral cyano-cyclopentadiene isomers has been discovered.^55^ These results show that, upon additional energy, (nitrogen containing) aromatic compounds are likely to isomerize to 5-membered ring structures prior to the fragmentation processes to different products.

### Astrophysical Implications and Conclusions

We have investigated the dissociative ionization of aniline using a combination of mass-spectrometry, infrared predissociation spectroscopy, and quantum chemical calculations. Five-membered ring structures, two H-cyano-CPD^+^ isomers, are found to be the dominant fragmentation products for the H-loss channel, contradicting earlier results that proposed six- or seven-membered ring species. The structural determination of the fragments provides important information on the dissociation processes of nitrogen containing aromatic molecules that are relevant in various astronomical environments.

In Titan’s atmosphere, the chemistry is initiated by the ionization and dissociation of the major atmospheric components, N_2_ and CH_4_. Both ionic and neutral reaction pathways coexist and drive the formation process of a thick haze of large organic molecules.^56−58^ Solar EUV (10–100 nm) radiation is the primary source of energy that is deposited in the atmosphere, and additional energy is provided by energetic electrons from Saturn’s magnetosphere and galactic cosmic rays.^59^ The solar radiation is therefore also sufficient for ionization and subsequent dissociation of larger (aromatic) organic species, possibly leading to cationic fragment structures. In addition, photoelectrons (energetic electrons of 24 eV) produced during photoionization of N_2_, in particular by the He II (30.4 nm) solar line, can induce further ionization and dissociation by electron impact ionization.^58^ The different masses observed by the Ion Neutral Mass Spectrometer (INMS) and Cassini Plasma Spectrometer (CAPS) onboard the Cassini spacecraft^12,17^ may therefore be a combination of both bottom-up formation pathways starting from N_2_ and CH_4_ and top-down dissociation products from larger organic species, both induced by energetic solar radiation and electrons.

In the mass spectrum of Titan’s atmosphere, a signal at *m*/*z* 92 is found and assigned to C_6_H_6_N^+^ as a closed shell cation of aniline. The anilino^+^ structure was hypothesized to contribute to C_6_H_6_N^+^ and to be the precursor for a chain reaction mechanism with small hydrocarbon molecules.^16,60^ From the spectroscopic determination here, we conclude that the anilino^+^ is not formed upon the hydrogen atom loss of aniline but that the most likely structure of the closed shell cation of aniline is that of the five-membered ring H-cyano-CPD^+^ isomers. This may have consequences on the reactivity of C_6_H_6_N^+^, and it would be interesting to investigate the reactivity of the isomeric species found here using both experimental and computational methods. Moreover, the mass of the HNC-loss fragment at *m*/*z* 66 that has previously been assigned to CPD^•+^ (C_5_H_6_^•+^) can be observed in the mass spectrum of Titan’s atmosphere.^17^ Different molecular species including nitrogen containing ones, such as C_4_H_4_N^+^, have also been considered for this *m*/*z* channel.^61^ On the contrary, for the *m*/*z* 65 and *m*/*z* 67 channels, only carbon containing species are accommodated in photochemical models.^62^ Considering the possible fragmentation of aniline in the upper atmosphere of Titan, the five-membered ring CPD^•+^ can contribute to the signal at *m*/*z* 66 and, analogously, a pure hydrocarbon structure with the molecular formula C_5_H_5_^+^, formed by subsequent hydrogen loss (as observed in the high-energy fragmentation spectrum in Figure 1b), can add to the feature with *m*/*z* 65. This shows that the dissociative ionization of six-membered ring structures, such as aniline, can yield five-membered ring structures with or without CN functional groups. The formation of products containing five-membered rings is not uncommon in fragmentation processes of larger aromatic molecules and has been observed for the dissociation of pure, nitrogen containing, and aliphatic PAHs.^20,63−65^

Five-membered rings are also possible building blocks to form larger organic molecules. Recently, the neutral cyclopentadiene cycle was detected in the cold molecular cloud TMC-1 with radio-astronomical observations.^7^ Moreover, the cyclopentadiene derivatives 1- and 2-ethynyl-cyclopentadiene and 1- and 2-cyano-cyclopentadiene have been detected in the same molecular cloud.^9,10,66^ Radical–neutral reactions between the small radical hydrocarbons CCH^•^ and CN^•^ and cyclopentadiene have been calculated to proceed exothermically and without a barrier.^10^ Also, proton transfer reactions can be important for the nitrogen containing cyano-cyclopentadiene molecules. The possibility of proton transfer reactions in TMC-1 has been shown for C_3_O and its protonated form HC_3_O^+^, which have both been detected in the same source with a high abundance of the protonated species (one-seventh of the neutral).^67^ To make a comparison, the proton affinities for 1-, 2-, and 3-cyano-CPD molecules have been calculated here (at the B3LYP-GD3/N07D level of theory) and are 824, 844, and 809 kJ/mol, respectively. The proton affinities of the cyano-CPD isomers are quite high and comparable to benzonitrile (812 kJ/mol^68^) and C_3_O (885 kJ/mol^69^), thereby indicating that protonated cyano-CPD^+^ isomers can be efficiently formed in molecular clouds such as TMC-1.

The observed protonated H-cyano-CPD^+^ isomers that have been formed here from the dissociative ionization of aniline are therefore also new important candidates for radio-astronomical searches in the interstellar medium. The rotational constants and dipole moment components of the three H-cyano-CPD^+^ isomers have been calculated and are shown in Table 1. Overall, the dipole moment components for the protonated cyano-CPD^+^ isomers are large and similar to the neutrals. For H-1-cyano-CPD^+^, we see a stronger c-type dipole moment due to the N–H group that is bent out-of-plane when compared to the neutral 1-cyano-CPD. To get an estimate on the agreement of calculated and experimental rotational constants, the experimental and calculated rotational constants of the neutral cyano-CPD isomers are shown as well. A good agreement between the calculated and experimental rotational constants is found.^9^ Laboratory microwave experiments are essential to improve the rotational constants and obtain the accurate line positions required for their astronomical detection.

**Table 1:** Comparison of Calculated and Experimental Rotational Constants and Dipole Moment Components of the Neutral Cyano-CPD Molecules and Their Protonated H-cyano-CPD^+^ Isomers^a^

	H-1-cyano-CPD^+^	1-cyano-CPD	1-cyano-CPD
molecular constants	calc	exp	calc
*A* (MHz)	8168.9	8352.981(10)	8345.8
*B* (MHz)	1839.9	1904.2522(2)	1896.4
*C* (MHz)	1517.6	1565.3652(2)	1560.2
μ_*a,b,c*_ (Debye)	2.7/0.2/0.8		4.6/0.3/0

	H-2-cyano-CPD^+^	2-cyano-CPD	2-cyano-CPD
molecular constants	calc	exp	calc
*A* (MHz)	8097.1	8235.592(14)	8219.3
*B* (MHz)	1835.2	1902.0748(3)	1895.5
*C* (MHz)	1509.9	1559.6472(2)	1555.1
μ_*a,b,c*_ (Debye)	4.2/1.1/0		5.0/0.6/0

	H-5-cyano-CPD^+^	5-cyano-CPD
molecular constants	calc	calc
*A* (MHz)	6208.4	6742.9
*B* (MHz)	2111.5	2091.7
*C* (MHz)	1798.9	1755.3
μ_*a,b,c*_ (Debye)	4.9/0/0.5	3.9/0/1.5

a The experimental rotational constants have been taken from Kelvin Lee et al.^9^ The rotational constants have been calculated at the B3LYP-GD3/N07D level of theory.

## Supporting Information Available

Mass spectrum of *m*/*z* 92 Ne tagging; saturation depletion scans (PDF)
