# Supplementary material for: Spectroscopic Detection of Cyano-Cyclopentadiene Ions as Dissociation Products upon Ionization of Aniline
Source: J Phys Chem A. 2022 May 5;126(19):2989–97. doi: 10.1021/acs.jpca.2c01429 (PMC9125686; doi:10.1021/acs.jpca.2c01429)
Supplement: Supplementary file 1 — jp2c01429_si_001.pdf [file jp2c01429_si_001.pdf]

Supporting Information:

Spectroscopic Detection of Cyano-Cyclopentadiene Ions as Dissociation Products upon Ionization of Aniline

Daniël B. Rap, Tom J. H. H. van Boxtel, Britta Redlich, Sandra Brünken*

Radboud University, Institute of Molecules and Materials, FELIX Laboratory, Toernooiveld 7, 6525 ED Nijmegen, The Netherlands, *E-mail: sandra.bruenken@ru.nl

Mass spectrum of m/z 92 Ne tagging

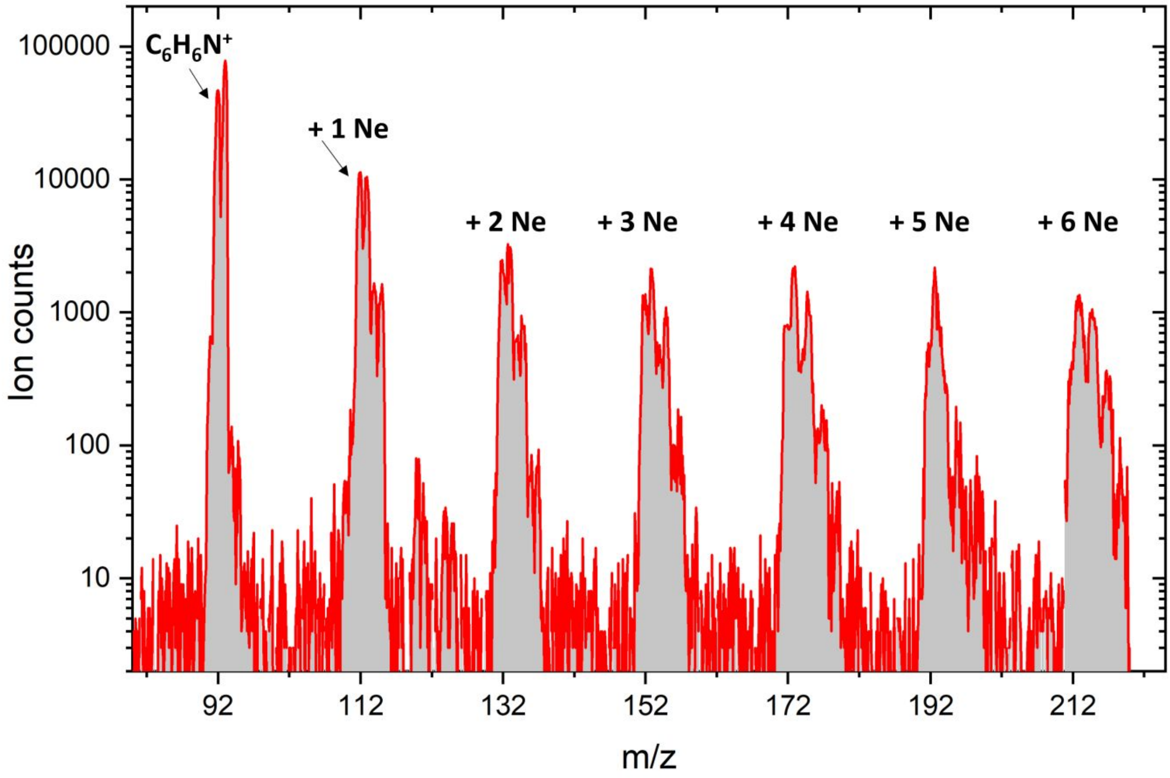

Figure S1: Mass spectrum of the Ne tagging of m/z 92 using the cryogenic 22-pole ion trap kept at a temperature of 6 K after a 50 ms long intense (number density around ~10^{14}–10^{15} cm^{-3}) He:Ne=2:1 pulse. Some contamination on m/z 93 admitted from the source is present.

Saturation Depletion scans

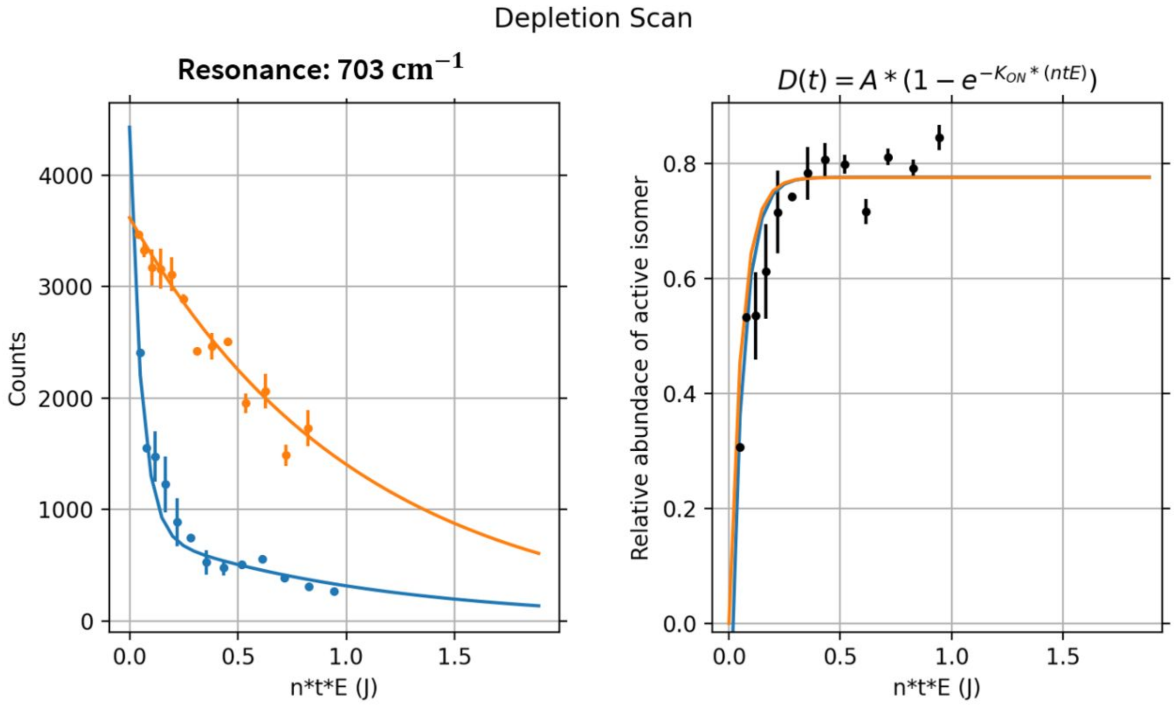

Figure S2: Saturation depletion scan of $C_6H_6N^+$-Ne (m/z 112) at the 703 cm^{-1} feature. The ion counts as a function of the number of infrared pulses is plotted for the on-resonance wavelength 703 cm^{-1} (blue, left panel) and off-resonance 804 cm^{-1} (orange, left panel). The relative abundance is fitted to ~78 % (right panel).

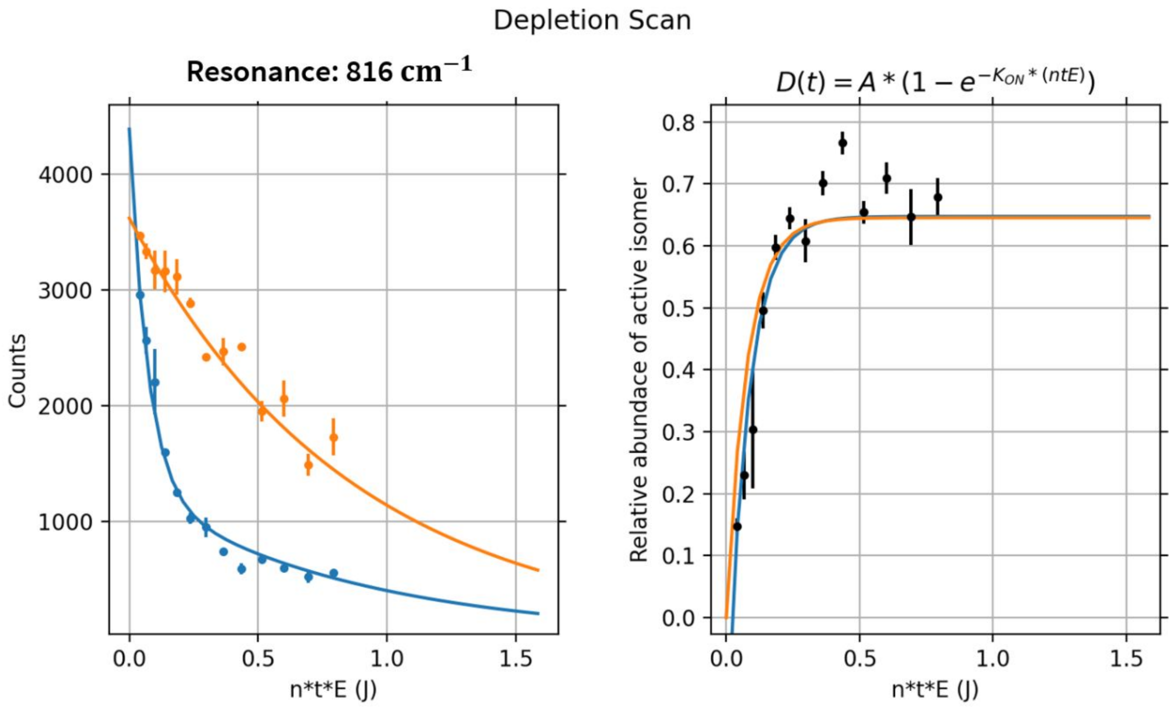

Figure S3: Saturation depletion scan of $C_6H_6N^+$-Ne (m/z 112) at the 816 cm^{-1} feature. The ion counts as a function of the number of infrared pulses is plotted for the on-resonance wavelength 816 cm^{-1} (blue, left panel) and off-resonance 804 cm^{-1} (orange, left panel). The relative abundance is fitted to ~65 % (right panel).
